# The Digital Library of Health Care Consultations and Simulated Health Care Student Teaching: Protocol for a Repository of Recordings to Support Communication Research

**DOI:** 10.2196/67910

**Published:** 2025-06-27

**Authors:** Elizabeth Ann Sturgiss, Kimberley Norman, Terry Haines, Katrina Long, Suzanne Nielsen, Jenny Sim, Aron Shlonsky, Brendan Shannon, Cylie Williams

**Affiliations:** 1 School of Primary and Allied Health Care Monash University Frankston Australia; 2 Faculty of Health Sciences and Medicine Bond University Robina Australia; 3 National Centre for Healthy Ageing Monash University Frankston Australia; 4 Department of Occupational Therapy Monash University Frankston Australia; 5 Eastern Health Clinical School Monash Addiction Research Centre Monash University Frankston Australia; 6 Department of Medical Imaging and Radiation Sciences School of Primary and Allied Health Care Monash University Clayton Australia; 7 Department of Social Work School of Primary and Allied Health Care Monash University Caulfield Australia; 8 Paramedicine School of Primary and Allied Health Care Monash University Frankston Australia

**Keywords:** health care communication, patient-physician relationship, community health care, medical education, health care education

## Abstract

**Background:**

Miscommunication in health care is a major source of poor health outcomes, complaints about health care professionals, and poor patient satisfaction. Recordings from real-life consultations provide valuable data for communication research and education. Additionally, recordings from simulation-based education of health care students can provide valuable data for health care education research.

**Objective:**

The Digital Library is a data repository supporting high-quality health care communication research. This is the single-source citation for all projects that use the Digital Library in Australia.

**Methods:**

This protocol outlines the logistics and consent process for recording and safely storing the recordings of health care consultations and simulation-based education. The processes are outlined for primary health care settings and health care educational settings as well as for health care narratives from consumers. The repository will be used to answer research questions about health care communication and provide a valuable resource for health care education.

**Results:**

Data collection for the Digital Library commenced in 2023 and is ongoing at the time of submission of this protocol. The Digital Library has been approved by Monash University’s Human Research Ethics Committee.

**Conclusions:**

The Digital Library will provide a national resource for the study of health care communication in community settings, general practice, and other environments. The health care narratives may be a valuable resource for sharing the patient perspective when living with different conditions. The research that uses this repository will be shared through regular academic channels as well as the community-based dissemination strategies of the National Centre for Healthy Ageing.

**International Registered Report Identifier (IRRID):**

DERR1-10.2196/67910

## Introduction

It is estimated that more than 18,000 deaths occur each year in Australia due to medical errors [1,2]. The Organisation for Economic Co-operation and Development estimates that 15% of hospital expenditure can be attributed to treating the consequences of patient safety failures [3]. Miscommunication in health care is known to impact and cause some medical errors [4], and is the second highest reason consumers raise complaints with health care regulators in Australia [5]. There are many and varied clinical situations where communication failures can occur, including poor handover between clinicians about a patient’s condition, inadequate consent processes, and poor rapport or bedside manner, particularly in the context of sensitive health discussions. A taxonomy of communication failures in the United Kingdom highlighted more than 50 categories of communication errors [6].

The harms of communication failures have been investigated in multiple areas of clinical practice and population groups, for example, communicating medication prescriptions with older adults following discharge from the hospital [7] and communicating with people with disability [8]. Poor communication between older adults who are culturally and linguistically diverse and health care workers has also been shown to lead to health disparities and discrimination, miscommunication, unmet needs, difficulties providing care [9], underreporting of symptoms [10], lower satisfaction with care [11], and increased use of potentially unnecessary diagnostic tests [12]. There are also communication issues that have been investigated that cut across population groups and clinical specialties, such as the investigation of unconscious biases influencing communication and that result in health inequities [13]. Recordings of health care consultations allow researchers a real-world view of communication as it happens, negating the effects of recall bias that occur with other research designs.

Several benefits have been identified by strengthening the communication skills of health professionals. Strong health care professional communication skills have been shown to improve health outcomes, particularly for pain [14], and positive communication can have placebo-like effects with improvements in patients’ anxiety, mood, and satisfaction [15]. Satisfaction with physician communication skills has been linked with better adherence to medications for hypertension [16] and improved patient-centered outcomes for older patients [17]. This points to communication skills training as a modifiable area for improving health care safety and quality and consumer satisfaction and engagement in their health care journey. Recordings of educational sessions that focus on communication skills are an opportunity to investigate best practices for learning.

The rise of virtual health care, including telehealth, has added another dimension to the investigation of how communication impacts health outcomes. Virtual care consultations have become a commonly used modality of health care delivery, particularly since the COVID-19 pandemic. Understanding the interaction between different communication techniques and this medium is important to promote safe and effective care [18]. While technology enables these consultations, research is only beginning to explore the unique communication dynamics between clinicians and patients in virtual settings. Understanding and refining these skills is essential, particularly given that virtual care consultations, often referred to as “black boxes” [19], occur behind closed doors, leaving much unknown about effective engagement strategies. With the emergence of virtual care, we need health care communication research that focuses on the complexity of communicating when cues such as body language, tone, and movement are missing.

Despite the impact of miscommunication in health care, there are few dedicated research infrastructures in the world to support research and education on real-life health care communication. In the Netherlands, there is a large repository held at Nivel, the Netherlands Institute for Health Services Research, that contains health care consultations from multiple disciplines [20]; in New Zealand, there is the Applied Research on Communication in Health Corpus at the University of Otago [21]; and in the United Kingdom, there is a repository of hundreds of general practice consultations called “One in a Million” at the University of Bristol [22].

The Digital Library, as a part of the National Centre for Healthy Ageing, will provide a window into real-life consultations in the Australian clinical environment and simulated health care consultations in education settings to support health care communication research and education. This research infrastructure aims to provide resources to improve consumer satisfaction and health care quality for health care consumers in Australia. This protocol outlines the process for data collection and storage, is the single-source citation for research that uses data from the Digital Library, and can also provide a framework for other groups internationally looking to establish a similar resource.

## Methods

### Overview

**Figure 1:**
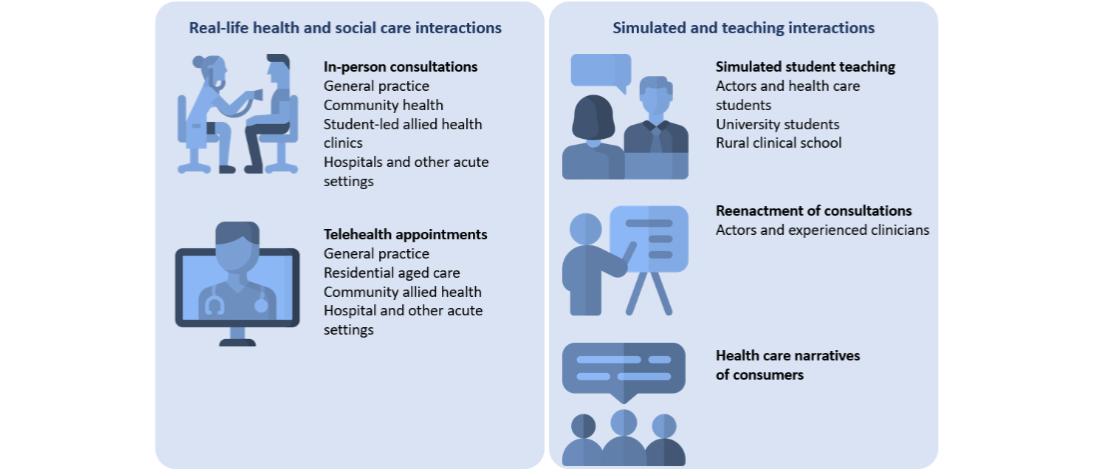
Types of video recordings to be housed in the Digital Library.

This protocol describes the recruitment, types of data, and logistics and storage for recordings being collected. This repository is a research infrastructure that will support research on health care communication practices to enhance patient health care safety and quality. The recordings can also be transcribed and used as scripts for recorded health care consultations with simulated actor patients and health care professionals. An overview of the types of video recording that will be housed in the Digital Library is provided in Figure 1.

### Setting

Health care settings include primary health clinics (eg, general practices, allied health clinics), health care educational settings (simulated teaching sessions between students, instructors, and trained actors), and student-led health care clinics that occur in-person and via virtual care.

### Patient and Public Involvement

Patients or the public were not involved in the design of this protocol but will be involved in future advisory groups that will shape the direction of the Digital Library and the research that is undertaken on the collected data.

### Types of Data in the Library

**Figure 2:**
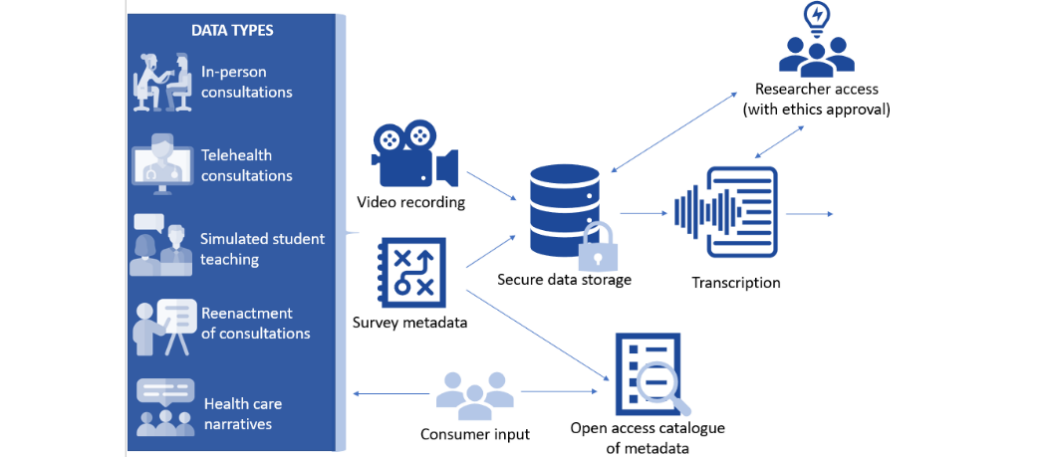
The logistics of data recording, storage, and access of the Digital Library.

The Digital Library will include in-person interactions and consultations, virtual consultations, health care narratives, and simulated interactions used in the teaching of health care professionals. Reenactments of these real-life scenarios will also be undertaken with simulated actor patients to enable wider dissemination across Australia while safeguarding privacy. The reenactments will be recorded on-site in Melbourne using anonymized scripts from the real-life consultations, with details changed to ensure confidentiality. An overview of the types of data and collection, storage, and retrospective access can be found in Figure 2.

As funding allows, investigators will add the following data to the Digital Library:

- Tailored metadata is ingested into a publicly available catalogue.
- Natural language processing is used to allow for increased discoverability of the data.

### Recruitment

#### Health Care Settings

Recruitment will occur in two stages: first, clinicians will be recruited, and then patients will be invited to participate on a recording day.

##### Recruiting Clinicians in Primary Health Settings

All types of clinicians working in primary health are eligible to be part of the Digital Library, including, but not limited to, general practitioners, practice nurses, nurse practitioners, and allied health clinicians. Investigators will use their professional networks and publicly available contact information. Health care professionals will be invited by email by the investigators through the following process:

- An initial letter or email of invitation will be sent to the clinician explaining the project and asking them to be involved. The email will include the information sheet for the project. The email will ask the participant to reply via email to arrange a time to discuss the project if interested.
- A phone call or email from the research team to the clinician will be undertaken 1-2 weeks after the letter/email. Clinicians who participate will be asked if they know of other clinicians who may be interested (snowball sampling).

Investigators will also post recruitment advertising on social media (Facebook, LinkedIn).

Research staff will first obtain written informed consent from health care professional participants. At this point, health care professional participants will be asked to complete a survey with demographic and professional experience details. Depending on the funding structure of the project, health care professionals may be provided an honorarium for their participation.

##### Recruiting Patients in Primary Health Settings

Patients who attend a participating clinic will be invited to be part of the Digital Library, with the intent to record their clinical interaction on that day. Any person who is acutely unwell, distressed, or unable to give written consent in English will not be approached to participate. All patients will be invited to participate when they attend the clinic, and when the patient is younger than 18 years, the parent/guardian will be invited. Reception staff will make potential patient participants aware that a study is occurring and provide them with an information sheet. Next, the research staff will approach the potential participant or parent to answer any questions they may have about the study and obtain written consent. When the patient is a child, they will be invited, provided a video about the project, and required to assent before participation. In the setting of virtual care, the health care professional will first explain that they are part of a research project. They will ask permission from the patient to record the telehealth consultation. They will also seek the patient’s permission to give their name and phone number to the research team. Using the patients’ contact details shared with their consent by their clinicians, the investigators will then contact the patient and explain the project in detail and obtain written consent from the patient by email. The patient can decline at this stage, and the recording will be deleted if written consent is not obtained or if the investigators cannot contact the patient.

Patients will be asked for consent to:

- Complete pre- and postconsultation surveys: The preconsultation survey will only be administered after the consultation in the telehealth setting. The patient will be emailed the surveys by the research team once they have received informed consent from the patient.
- Video recording of their consultation
- Permission to keep their video recording on a secure server as part of a library of consultations: These consultations will only be used for research, and any further study outside of this remit will require ethics approval. Research requests to use the Digital Library data require approval from the Digital Library governance team as well as appropriate ethics approval.

#### Simulated Teaching Sessions

##### Overview

Education sessions that involve simulation or role-plays will be eligible for recording. Our team of investigators has connections with the health professional training programs at our university. All health care disciplines are eligible for recording, including, but not limited to, medicine, podiatry, physiotherapy, paramedicine, occupational therapy, social work, and radiation therapy. Consent will occur in two steps.

##### Recruitment of Educators in University Health Care Programs

Educators will be invited by email from their public staff profile. An initial email of invitation will be sent explaining the project, and it will include the explanatory statement. Educators will be asked to reply by email to permit us to contact them to arrange a meeting to discuss the project in detail. Educators who participate will be asked if they know of other educators who may be interested in participating. The Digital Library will also advertise in professional newsletters, on social media, and using flyers on campus.

##### Recruitment of Health Care Students

Students will be approached in person outside the classroom before their simulated teaching session. Research assistants will hand out an explanatory statement, verbally explain the project, and obtain written consent. For online teaching and student-led telehealth clinics, the course coordinator or lecturer associated with the teaching unit or clinic will send the students the project information and ask them to contact the research team if they are interested. This will preserve students’ privacy as students will give their details to the research team directly.

#### Health Care Narratives

Investigators will collect health narratives from a diverse range of health care consumers with their informed written consent. Health care consumers will be invited to narrate their own story and experience of health care for inclusion in the Digital Library. Participants are invited via an online consumer platform at the National Centre for Healthy Ageing as well as public announcements at National Centre for Healthy Ageing events. There are no exclusion criteria for health care narrative participants. All underrepresented or marginalized population groups (including but not limited to Indigenous and First Nations peoples, people living with disability, people who speak English as a second language, and those who are gender diverse) are encouraged to participate. Purposeful recruitment strategies will be undertaken to reduce any equity-related communication barriers to participation. This includes, for example, partnering with community organizations to support safe and inclusive engagement and participation.

Researchers will use an unobtrusive interviewing style and ask broad questions only (Multimedia Appendix 1). Investigators will recruit participants via professional networks, contacts via the health care teaching programs, and community advertisements. Consumers may be offered an honorarium depending on the funding available in appreciation of their time and effort. Recordings will be made in a setting that is convenient for the participant. Consumers will be able to choose how they would like their narrative to be accessed: for research purposes, for educational purposes (eg, to be shown in teaching sessions), or for community viewing (eg, on a public website).

### Data Collection

In health settings, investigators will collect data from:

- Clinician survey: The clinician will be asked to fill in a survey that includes demographic and professional experience details before the day or early on the day of recording (Multimedia Appendix 1).
- Health care consumer surveys (pre- and postconsultation): Health care consumers will be asked to complete a survey before their consultation detailing their demographics, reason for visit, and how long they have known the clinician. Post consultation, they will be asked to complete a survey detailing their consultation experience, including whether they felt respected, felt listened to, and had enough time (Multimedia Appendix 1).
- Video recording of the consultation: A camera will be placed in the consultation room aimed at the full face of the clinician without the patient in view, but with both voices being recorded. No physical examination that takes place away from the camera will be video recorded. The audio recording will remain on for the full consultation.

In educational settings, data will be collected from:

- Educator survey including demographic and professional experience details (education and clinical; Multimedia Appendix 1).
- Video recording of the educational setting

At this stage, students will not complete a survey. This may be added later should it prove to be useful for research purposes.

In health care narrative settings, investigators will collect data from:

- Participant demographic survey (Multimedia Appendix 1).
- Video recording of the participant sharing their narrative in their home, a private space at a Monash University campus, or via a Zoom (Zoom Communications) call

### Data Storage and Security

#### Storage

This protocol mediates between the opposing forces of the need for reliable, accessible data for research and training, and the need to protect the privacy of participants. Table 1 illustrates our approach to the accessibility, privacy, and rediscoverability of the video recordings.

The Digital Library will be accessible while respecting the privacy and confidentiality of participants. It will also be built to embody the findable, accessible, interoperable, and reusable (FAIR) principles, and our activities will consider the collective benefit, authority to control, responsibility, and ethics (CARE) principles of data storage [23]. All participants provide informed consent that their data will be stored indefinitely and potentially accessed in the future. This is justified and ethically approved, as the purpose of the Digital Library is to grow a data resource that can provide real-world insights into the changes to health consultation interactions across time, similar to Nivel in the Netherlands. Participants are made aware through the consent process and encouraged to contact the Digital Library if they wish their data to be removed moving forward. There is no re-consent process as this is the responsibility of the participant. Participant data will be removed immediately following any such request. If participants do not consent in the first place, their data is not stored in the Digital Library.

The data will be stored indefinitely and discoverable in three tiers:

1. Raw data of the recorded real-world consultations: accessible through in-person, private screening pods on-site at Monash University within the National Centre for Healthy Ageing, or after relevant ethics approvals
2. Transcripts and reenactments: accessible off-site after ethics approvals
3. Curated metadata: open access, searchable catalog that allows researchers worldwide to know what is available in the Digital Library and plan their research accordingly

This approach enables us to work within the FAIR and CARE principles of research data access, allowing the research platform to be used by as many people as possible while preserving the privacy of participants (Table 1).

**Table 1:** The types of data in the Digital Library and accessibility across Australia.

Recording types	Level of sensitivity	Data access		
		Video recordings	Deidentified transcriptions	Catalog of metadata
In-person consultations	High	On-site with ethics approval	Off-site with ethics approval	Open access
Telehealth appointments	High	On-site with ethics approval	Off-site with ethics approval	Open access
Simulated student teaching	Medium	On-site with ethics approval	Off-site with ethics approval	Open access
Reenactment of consultations	Low	Open access	—^a^	Open access
Health care narratives	Low	Open access	—	Open access

^a^Not applicable.

#### Video Recordings

The recordings will be stored on a separate on-site computer that can only be accessed by a primary investigator and research staff who specifically require access. Monash University’s Information Technology Department will secure the computer so that it is the only device able to access the recordings. The computer access will be housed in the National Centre for Healthy Ageing at the Peninsula Campus of Monash University. The real-life recordings will only be accessed for research purposes.

#### Paper-Based Surveys

All paper surveys will be scanned and stored on a secure, password-protected network drive. Once stored, the paper copies will be destroyed. The survey answers will be transferred to an electronic file that is maintained on a secure, password-protected network drive.

### Confidentiality

The participants’ names will be linked to a unique identifier. This will be kept in an electronic file, separate from the recordings and paper-based surveys, and maintained on a secure, password-protected network drive. This information will be kept so that the patient can request deletion of their video at any time in the future via email or phone call to the research team. If the video has been used for research that is in progress or completed, the video cannot be removed from the research. However, the video will be destroyed so that it cannot be used in future research.

### Access to the Digital Library for Researchers

Access to the data aligns with the policies of the National Centre for Healthy Ageing and Monash University, and ethical and legal requirements. Researchers can contact the Digital Library team using an expression of interest form on the Digital Library website, which includes details of the project methodology, research aims, specific data they are requesting access to, and proof of ethics approval from their governing body. This application is then assessed by our wider governance team for approval. The governance team consists of experts in primary and allied health care, partner organizations, and marginalized community group representatives who are trained in bias influence, power, and equity issues in research. The approval process first consists of an application assessment from the governance team review committee to determine the sensitivity level of the data requested. Low sensitivity data can be approved at this stage, and mid-to-high sensitivity data is then assessed by the governance team. The governance team will assess the application against a set of standard requirements. This includes ethical considerations, appropriateness of the research, and whether the application is aligned with community needs. The governance team will assess applications independently and then discuss results as a team to approve or decline data release.

If approved, the researchers sign a confidentiality agreement and are given access to the required set of data that they need on-site at the Peninsula Campus on a secure-patched computer.

## Results

The Digital Library has ethics approval from Monash University’s Human Research Ethics Committee. It has received initial funding from the National Centre for Healthy Ageing and began collecting data in 2023. At the time of protocol submission, data collection from health care settings, education settings, and health care narratives was ongoing.

## Discussion

Over time, the Digital Library will house the largest collection of health care interactions in Australia, providing research infrastructure to accelerate new knowledge in the areas of miscommunication in health care, communication techniques and aids, and clinical handover, including from clinician to patient.

The Digital Library will support methods such as conversation analysis, linguistics, multimodal video analysis of speech and nonverbal aspects of communication, and direct consumer insights. Examples of research that have used the pilot dataset include:

- A conversation analysis of general practitioner (GP) consultations to investigate GP and patient laughter when discussing lifestyle [24]
- An interview study while watching their recorded consultation with patients from low socioeconomic groups on what makes a good GP consultation [25]
- A secondary analysis of video recordings to examine how GPs discuss weight in consultations [26]
- An interview study where allied health professionals (physiotherapists and podiatrists) discuss lower limb conditions that result in chronic pain (in progress)
- A student training resource where a podiatrist is performing a nail surgical procedure and talking to the patient during and after the procedure (in progress)
- A simulation of initial domestic violence assessment and safety planning for social work trainees (planned)

It is also possible to conduct experimental studies using innovative communication techniques and real-life observational data [15,27]. The data from the Digital Library will support evidence-based health care communication research to ultimately improve health care quality and patient satisfaction. The new knowledge generated via the library will be relevant for clinician training and professional development, health care models of access, and consumer-driven health care improvements.

## Appendix

Multimedia Appendix 1 Study surveys.

## Abbreviations

CARE: collective benefit, authority to control, responsibility, and ethics
FAIR: findable, accessible, interoperable, and reusable
GP: general practitioner
