# Supplementary material for: The Digital Library of Health Care Consultations and Simulated Health Care Student Teaching: Protocol for a Repository of Recordings to Support Communication Research
Source: JMIR Res Protoc. 2025 Jun 27;14:e67910. doi: 10.2196/67910 (PMC12254701; doi:10.2196/67910)
Supplement: Multimedia Appendix 1 [file resprot_v14i1e67910_app1.pdf]

Clinician survey

Please enter your first name and surname below

[ ]

How old are you?

☐ <35 years ☐ 35 -44 years ☐ 45-54 years ☐ 55 years and over
☐ Prefer not to say

What is your gender?

☐ Female ☐ Male ☐ Non-binary/ third gender

☐ Prefer to self-describe ____________________

☐ Prefer not to say

How many years have you been working in general practice in total?

☐ <2 years ☐ 2-5 years ☐ 6-10 years ☐ 11-19 years

☐ 20 years and over ☐ Prefer not to say

Are you:

☐ FRACGP

☐ ACCRM

☐ FARGP

☐ Non-vocationally registered GP

☐ GP registrar

☐ Other ______________________________________

☐ Prefer not to say

What country were you born in?

☐ Country: ______________________________

☐ Prefer not to answer

How would you describe your cultural background?

☐ Cultural Background: ______________________________

☐ Prefer not to answer

Are you of Aboriginal or Torres Strait Islander origin?

☐ Yes, Aboriginal

☐ Yes, Torres Strait Islander

☐ Yes, both Aboriginal and Torres Strait Islander

☐ No

☐ Prefer not to say

Do you use a language other than English at home?

☐ Yes (please specify which language)

____

☐ No

Number of direct patient care hours per week:

☐ less than or equal to 10 hours

☐ 11 – 20 hours

☐ 21 – 40 hours

☐ 41 – 60 hours

☐ 61+ hours

☐ Prefer not to say

Place of graduation from medical school:

☐ Australia

☐ Asia

☐ UK/Ireland

☐ Africa and Middle East

☐ Europe

☐ New Zealand

☐ Other ____________________________

☐ Prefer not to say

Consultations in languages other than English:

☐ none

☐ less than 25%

☐ 25 - 50%

☐ more than 50%

☐ Prefer not to say

Healthcare Educator Survey

ID: .... (digital library office uses only)

Educator Name

____

How old are you?

☐ <35 years ☐ 35 -44 years ☐ 45-54 years ☐ 55 years and over ☐ Prefer not to say

What is your gender?

☐ Female ☐ Male ☐ Non-binary/ third gender

☐ Prefer to self-describe ___________________

☐ Prefer not to say

What country were you born in? _______________________

☐ Prefer not to say

How would you describe your cultural background?

____

☐ Prefer not to say

Are you of Aboriginal or Torres Strait Islander origin?

☐ Yes, Aboriginal

☐ Yes, Torres Strait Islander

☐ Yes, Both Aboriginal & Torres Strait Islander

☐ No

☐ Prefer not to say

Do you speak another language other than English at home?

☐ Yes, please specify ______________________________

☐ No

How many years have you been working in health education in total?

☐ <2 years ☐ 2-5 years ☐ 6-10 years ☐ 11-19 years

☐ 20 years and over ☐ Prefer not to say

Please describe the discipline that your health educator role is affiliated with? (ie Social work, Physiotherapy, Occupational Therapy, Paramedicine, Medicine)

☐ Please describe: _________________________________

☐ Prefer not to say

Number of direct educator employment hours per week:

☐ less than or equal to 10 hours

☐ 11 – 20 hours

☐ 21 – 40 hours

☐ 41 – 60 hours

☐ 61+ hours

☐ Prefer not to say

Thank you for taking the time to complete this survey.

Please return once completed to digital.library@monash.edu

MONASH UNIVERSITY DIGITAL LIBRARY

Healthcare Narrative Demographic Survey ID: ….

Participant Name

____

How old are you?

☐ <35 years ☐ 35 -44 years ☐ 45-54 years ☐ 55 years and over
☐ Prefer not to say

What is your gender?

☐ Female ☐ Male ☐ Non-binary/ third gender
☐ Prefer to self-describe ____________________
☐ Prefer not to say

What country were you born in? ________________________

☐ Prefer not to say

How would you describe your cultural background?

☐ Prefer not to say

____

Are you of Aboriginal or Torres Strait Islander origin?

☐ Yes, Aboriginal
☐ Yes, Torres Strait Islander
☐ Yes, Both Aboriginal & Torres Strait Islander
☐ No
☐ Prefer not to say

Do you speak another language other than English at home?

☐ Yes, please specify ___________________________________
☐ No

How many years have you been working in health education in total?

☐ <2 years ☐ 2-5 years ☐ 6-10 years ☐ 11-19 years
☐ 20 years and over ☐ Prefer not to say

Please describe the main healthcare topic/s included in your healthcare experience you are sharing today (ie health condition, health context, health professional interaction etc)

☐ Please describe: ______________________________________

☐ Prefer not to say

Thank you

Appendix: Healthcare Narrative Question Guide

<u>Before Recording</u>

Introductions (participant and researcher)

Explain the project (Explanatory Document)

Explain the participants rights again (they can stop at any time, no questions asked)

Explain what your role is in the recording and what the participant can expect from researcher

For example:

- The researcher will listen only
- Ask questions related to the story as prompts
- Not offer any opinion/ advice/ perspective
- Support the participant if they want to stop, pause or withdraw from sharing their experience
- Advise them that the researcher will remind them when there is 5 minutes until 30 minutes as a marker for their time they wish to spend speaking about their experience.

Answer any questions the participant has before recording

Make sure consent is signed

Ensure Demographic Survey is completed

Turn on Video Recording – ask consent to start the recording and record this verbal consent.

When the participant is ready, begin with the first question

<u>Questions</u>

1. Please tell me about your healthcare experience

   Prompts (for researcher)

   - *How did that make you feel?*
   - *How was the experience with your health care professional?*

2. Is there anything else you would like to share with us about your healthcare experience?

Be mindful

- Not to give any advice that you are not qualified to do so
  For example, do not offer any medical advice or psychological advice. You can offer them details of psychological services who can help them (Beyond Blue) or to contact their GP.

Thank the participant for their time

Stop recording

Patient survey
PRIOR to CLINICIAN appointment

ID:…………………

How old are you?

☐ Less than 18 years ☐ 18-24 years ☐ 25-34 years ☐ 35-44 years
☐ 45-54 years ☐ 55-64 years ☐ 65-74 years ☐ 75-84 years
☐ 85 years and over ☐ Prefer not to say

What is your gender?

☐ Woman or Female ☐ Man or Male ☐ Non-binary
☐ I use a different term (please specify) ________________
☐ Prefer not to answer

What country were you born in?

☐ Name of country:

☐ Prefer not to answer

How would you describe your cultural background?

☐ Cultural Background:

☐ Prefer not to answer

Are you of Aboriginal or Torres Strait Islander origin?

☐ Yes, Aboriginal
☐ Yes, Torres Strait Islander
☐ Yes, both Aboriginal and Torres Strait Islander
☐ No
☐ Prefer not to say

Do you use a language other than English at home?

☐ Yes (please specify which language)

☐ No
☐ Prefer not to say

Do any of these apply to you? Please tick all that apply.

☐ Unemployed and looking for work
☐ Receive government pension
☐ Healthcare card holder
☐ Live in a low-income household

How long have you seen the clinician you are seeing today?

☐ Meeting them today for the first time
☐ This is second appointment I have ever had with them
☐ Less than one year (more than one appointment before)
☐ About one –five years
☐ More than five years
☐ Prefer not to say

Is the CLINICIAN you are seeing today your preferred CLINICIAN?

☐ Yes
☐ No
☐ Prefer not to say

What are the reasons for your consultation today? Tick all that apply

☐ To find out what is wrong / get a diagnosis
☐ For reassurance
☐ To get the results of test / investigations
☐ For treatment (prescriptions, procedures)
☐ For a routine check
☐ For review
☐ To ask for a referral
☐ Other - ______________
☐ Prefer not to say

Are you attending your consultation alone or with a support person?
☐ Alone
☐ With a support person
☐ Prefer not to say

Do you have any of the chronic conditions from the list below? (please tick all that apply)

☐ High blood pressure
☐ Depression or anxiety
☐ Musculoskeletal condition
☐ Arthritis
☐ Osteoporosis
☐ Chronic lung condition (e.g. asthma, COPD, chronic bronchitis)
☐ Heart disease (angina, ischaemic heart disease, peripheral vascular disease)
☐ Heart failure
☐ Stroke or TIA
☐ Reflux, or gastric ulcer, or peptic ulcer
☐ Bowel disease (IBS, ulcerative colitis, Crohn's disease, diverticulosis, diverticulitis)
☐ Chronic hepatitis
☐ Diabetes (type 1 or 2)
☐ Thyroid disease
☐ Cancer in the last 5 years (including melanoma, but excluding other skin tumours)
☐ Kidney disease or kidney failure
☐ Chronic urinary tract infections
☐ Dementia or Alzheimer's disease
☐ High cholesterol
☐ Obesity
☐ Other …………………………………………..
☐ Prefer not to say
☐ No chronic condition

If yes to any of the conditions, how long ago was the first condition diagnosed?

☐ < 1 year ☐ 1 – 5 year ☐ 5 – 10 year ☐ > 10 year

AFTER CLINICIAN appointment

Only the research team will have access to your answers. Your CLINICIAN WILL NOT have access to your answers.

Below is a list of statements describing how people might interact with their CLINICIAN. Please tick your response under the statement.

1. **As a result of seeing my CLINICIAN, I am clearer as to how I can look after my health and wellbeing.**

☐ Strongly disagree ☐ Disagree ☐ Neither agree or disagree ☐ Agree ☐ Strongly agree

2. **What I am doing with my CLINICIAN gives me new ways of looking at my health and wellbeing.**

☐ Strongly disagree ☐ Disagree ☐ Neither agree or disagree ☐ Agree ☐ Strongly agree

3. **I believe my CLINICIAN cares about me.**

☐ Strongly disagree ☐ Disagree ☐ Neither agree or disagree ☐ Agree ☐ Strongly agree

4. **My CLINICIAN and I work together on setting goals for looking after my health and wellbeing.**

☐ Strongly disagree ☐ Disagree ☐ Neither agree or disagree ☐ Agree ☐ Strongly agree

5. **My CLINICIAN and I respect each other.**

☐ Strongly disagree ☐ Disagree ☐ Neither agree or disagree ☐ Agree ☐ Strongly agree

6. **My CLINICIAN and I are working towards health goals that we both agree on.**

☐ Strongly disagree ☐ Disagree ☐ Neither agree or disagree ☐ Agree ☐ Strongly agree

7. **I feel that my CLINICIAN understands me.**

☐ Strongly disagree ☐ Disagree ☐ Neither agree or disagree ☐ Agree ☐ Strongly agree

8. My CLINICIAN and I agree on what is important for me to do to look after my health and wellbeing.

☐ Strongly disagree ☐ Disagree ☐ Neither agree or disagree ☐ Agree ☐ Strongly agree

9. Even though I may do things that my CLINICIAN does not advise or suggest, I know they still care about me.

☐ Strongly disagree ☐ Disagree ☐ Neither agree or disagree ☐ Agree ☐ Strongly agree

10. I feel the things I do with my CLINICIAN will help me to achieve my health goals.

☐ Strongly disagree ☐ Disagree ☐ Neither agree or disagree ☐ Agree ☐ Strongly agree

11. My CLINICIAN and I have a shared understanding of what I need to do to look after my health and wellbeing.

☐ Strongly disagree ☐ Disagree ☐ Neither agree or disagree ☐ Agree ☐ Strongly agree

12. I think we're doing the right things for my health and well-being.

☐ Strongly disagree ☐ Disagree ☐ Neither agree or disagree ☐ Agree ☐ Strongly agree

Did the CLINICIAN listen carefully to you?

☐ Yes

☐ No

☐ Prefer not to say

Did they show respect for what you had to say?

☐ Yes

☐ No

☐ Prefer not to say

Did they spend enough time with you?

☐ Yes

☐ No

☐ Prefer not to say

Was your consultation today about a chronic condition?

☐ Yes

☐ No

☐ Prefer not to say

☐ I do not have a chronic condition

Did you discuss pain with your clinician today?

☐ Yes, pain that I've had for less than 3 months

☐ Yes, pain that I've had for more than 3 months

☐ No

☐ Prefer not to say

☐ I do not have any pain

Please tick the box that best represents your response.

1. **To what extent was your main problem(s) discussed today?**
   ☐ Completely ☐ Mostly ☐ A little ☐ Not at all

2. **How satisfied were you with the discussion of your problem?**
   ☐ Completely ☐ Mostly ☐ A little ☐ Not at all

3. **To what extent did the CLINICIAN listen to what you had to say?**
   ☐ Completely ☐ Mostly ☐ A little ☐ Not at all

4. **To what extent did the CLINICIAN explain this problem to you?**
   ☐ Completely ☐ Mostly ☐ A little ☐ Not at all

5. **To what extent did you and the CLINICIAN discuss your respective roles? (who is responsible for making decision and who is responsible for what aspects of your care?)**
   ☐ Completely ☐ Mostly ☐ A little ☐ Not at all

6. **To what extent did the CLINICIAN explain treatment?**
   ☐ Completely ☐ Mostly ☐ A little ☐ Not at all

7. **To what extent did the CLINICIAN explore how manageable the treatment would be for you?**
   ☐ Completely ☐ Mostly ☐ A little ☐ Not at all

8. **How well do you think your CLINICIAN understood you today?**
   ☐ Completely ☐ Mostly ☐ A little ☐ Not at all

9. **To what extent did the CLINICIAN discuss personal or family issues that might affect your health?**
   ☐ Completely ☐ Mostly ☐ A little ☐ Not at all

1.Please rate the following statements about today's consultation. Please tick one box for each statement and <u>answer every statement</u>.

How was the doctor at …	**Poor**	**Fair**	**Good**	**Very Good**	**Excellent**	**Does Not Apply**
**1. Making you feel at ease……** *(being friendly and warm towards you, treating you with respect; not cold or abrupt)*	☐	☐	☐	☐	☐	☐
**2. Letting you tell your "story"……** *(giving you time to fully describe your illness in your own words; not interrupting or diverting you)*	☐	☐	☐	☐	☐	☐
**3. Really listening ……** *(paying close attention to what you were sayings; not looking at the notes or computer as you were talking)*	☐	☐	☐	☐	☐	☐
**4. Being interested in you as a whole person …** *(asking/knowing relevant details about your life, your situation; not treating you as "just a number")*	☐	☐	☐	☐	☐	☐
**5. Fully understanding your concerns……** *(communicating that he/she had accurately understood your concerns; not overlooking or dismissing anything)*	☐	☐	☐	☐	☐	☐
**6. Showing care and compassion….** *(seeming genuinely concerned, connecting with you on a human level; not being indifferent or "detached")*	☐	☐	☐	☐	☐	☐
**7 . Being Positive……** *(having a positive approach and a positive attitude; being honest but not negative about your problems)*	☐	☐	☐	☐	☐	☐
**8. Explaining things clearly……** *(fully answering your questions, explaining clearly, giving you adequate information; not being vague*	☐	☐	☐	☐	☐	☐
**9. Helping you to take control……** *(exploring with you what you can do to improve your health yourself; encouraging rather than "lecturing" you)*	☐	☐	☐	☐	☐	☐
**10. Making a plan of action with you …** *(discussing the options, involving you in decisions as much as you want to be involved; not ignoring your views)*	☐	☐	☐	☐	☐	☐
